# Optical diffraction tomography using a self-reference module

**DOI:** 10.1364/BOE.545296

**Published:** 2024-12-04

**Authors:** Zhengyuan Tang, Julianna Winnik, Bryan M. Hennelly

**Affiliations:** 1 Department of Electronic Engineering, Maynooth University, Maynooth, Co. Kildare, Ireland; 2 The Institute of Micromechanics and Photonics, Faculty of Mechatronics, Warsaw University of Technology, Warsaw, Poland; 3 Department of Computer Science, Maynooth University, Maynooth, Co. Kildare, Ireland

## Abstract

Optical diffraction tomography enables label-free, 3D refractive index (RI) imaging of biological samples. We present a novel, cost-effective approach to ODT that employs a modular design incorporating a self-reference holographic capture module. This two-part system consists of an illumination module and a capture module that can be seamlessly integrated with any life-science microscope using an automated alignment protocol. The illumination module employs a galvo-scanner system, providing precise control over the angular illumination, while the capture module utilises the principle of self-reference off-axis holography. The design has a compact form factor, simple alignment, and reduced cost. Furthermore, our system offers the capability to switch between two imaging modalities, ODT and real-time synthetic aperture digital holographic microscopy (SA-DHM), a unique feature not found in other setups. Experimental results are provided using a kidney cancer cell line. Experimental results are provided using a kidney cancer cell line.

## 1. Introduction

Optical diffraction tomography (ODT) is a label-free microscopic imaging method that enables visualisation of the three-dimensional (3D) refractive index (RI) variation within a sample. ODT shares some similarities with Synthetic-Aperture Digital Holographic Microscopy (SA-DHM) in that they both require a series of holograms to be recorded over a range of oblique illuminations; while SA-DHM simply stitches the frequency bands together to form a synthetic aperture, ODT is also based on aggregating these bands together using a complex reconstruction process that calculates the 3D scattering potential function. The underlying theory of ODT was first proposed by E. Wolf in 1969 [1] and since then various optical implementations and numerical reconstruction processes have been proposed. [2–4]. There are two fundamental types of ODT systems: sample rotation ODT [5–7] and illumination scanning ODT [8–11]; the former uses a sample rotater to adjust the angle of the sample relative to a fixed illumination angle and multiple projections are used to reconstruct the 3D RI map. However, this approach suffers from slow acquisition times and alignment issues. Illumination scanning ODT is far more common and has recently been enhanced by numerical reconstruction methods that can mitigate the problem of the missing ‘apple-core’ in the 3D potential function using nonnegativity constraint optimisation methods [12,13]. Scanning illumination ODT has been demonstrated to be a powerful method for the investigation of biological cells and cellular dynamics [14,15].

Various methods of implementing scanning ODT have been proposed in the literature on the basis of interferometry and at this time at least two such methods are commercially available. On the illumination side, the oblique scanning illumination angle can be provided by a galvo-scanner [14–17], a spatial light modulator or digital micro-mirror device [18–20], or a rotating mirror system [21]. Most implementations make use of a single laser source that is split into object and reference paths before being recombined at the camera. Typically the reference wave is incident on the camera at a fixed off-axis angle, which separates the complex twin images in Fourier space; each captured aperture can be filtered from the Fourier domain via a discrete Fourier transform (DFT) operation and mapped onto 3D Fourier space for tomographic reconstruction. Recently, a number of self-reference methods have been proposed by the Park group, whereby the object wavefield can be used to generate a reference wavefield just before the camera. This type of approach is more easily attached as an add-on to an existing microscope. In Ref. [11] a Ronchon polarizer is positioned just before the camera, which generates two copies of the object wave with one at an off-axis angle. While simple, this method requires that object field to be sparse in the area of overlap. In [14–17], an implementation is proposed that overcomes this limitation; once again the object wavefield is used to generate the reference wavefield but in this case a diffraction grating produces a copy of the object wave which is then filtered by a pinhole in order to generate a plane wave reference. While powerful, this method suffers from a large form factor requiring a 12−f imaging system before the camera. Furthermore, a second 2D galvo-scanner is required at the capture side in order to redirect the zero frequency through a single static pinhole filter as the illumination angle changes.

In this paper, we propose a self-reference module that is significantly smaller in size and cost when compared with [14]. Furthermore, the module can easily be adapted to record a real-time SA-DHM. No previous implementation of ODT has been able to merge these modalities. We note that the ODT system presented here is based on an extension of our recently proposed real-time SA-DHM system in Ref [22].

## 2. Optical system

**Fig. 1.**
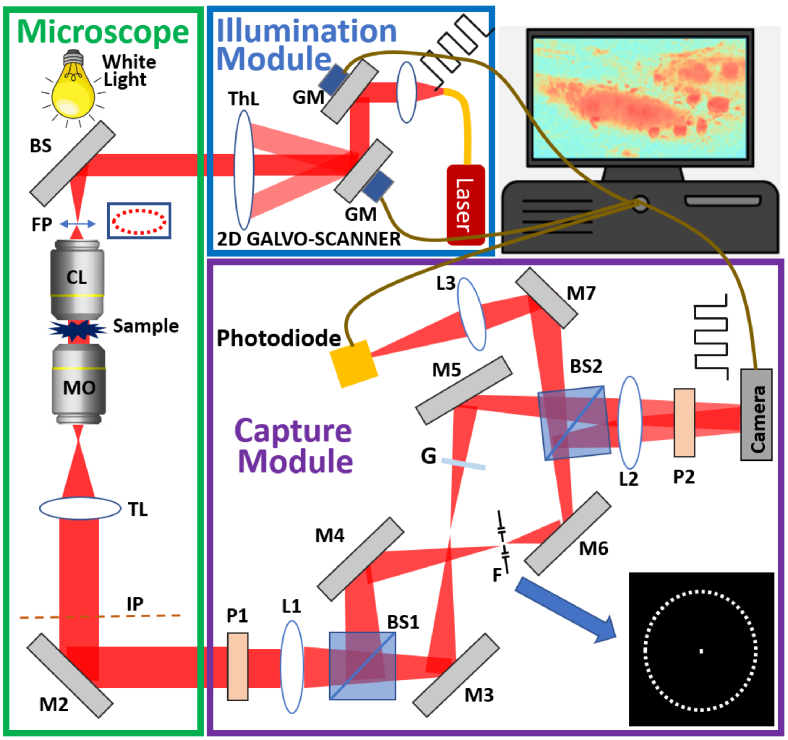
The structure of our self-reference ODT setup that includes an illumination module, a microscopy and a capture module: GM, Galvo-mirrors; ThL, theta lens; FP, filter plane; CL, condenser lens; MO, microscope objective; TL, Tube lens; IP, image plane; M, mirror; L, lens; BS, beamsplitter; F, annular-pinholes filter; G, transparent glass that has the same thickness with the pinholes filter; P, polarizer.

The structure of the proposed self-reference ODT setup is shown in Fig. 1 and is divided into three parts: an illumination module, a microscope, and a capture module. This modular approach is made possible by the self-reference design, which facilities decoupling of the illumination and capture modules. The ability to attach these relatively low-cost modules to any existing life-science microscope is an important feature of the design.

The illumination module uses a low cost 635 nm laser diode source with single mode fiber output (Thorlabs; HLS635); the output of the fiber (Thorlabs; SM600) is collimated using a plano-convex lens (Thorlabs; LA1131-A) with focal length 5 cm. The resultant beam is input to a two-dimensional galvo-scanning mirror system (Thorlabs GVS012) with repeatability 15 μrad , which comprises of two voltage controlled galvonometers and a servo driver board. This board is driven by the analog output of a digital acquisition (DAQ) card (National Instruments Corp; USB-6003); the card acts as a digital-to-analog converter that is controlled by Labview Software. This system steers the beam to an F-theta lens (Thorlabs FTH254-1064) with focal length 254 mm, which focuses the light to a spot at back focal plane of the microscope condenser lens, denoted in the figure as the filter plane (FP). The position of the spot is programmed to move to a sequence of 180 discrete positions in an annular pattern in the filter plane, which is discussed in further detail below.

The illumination module is attached to a bespoke microscope comprising a microscope objective (Olympus; MPlan 40x) with numerical aperture $NA_{MO}=0.85$ and tube lens (Olympus; SWTLU-C, f=180mm). The condenser lens (Nikon; Plan Apo 20x, 0.75) is mounted on a manual translation stage (Thorlabs; MT1-M), which is adjusted to ensure Kohler illumination. The sample is mounted on an electronic translation stage (ASI; MS-2000, LX-4000) and the MO is also mounted on an electronic translation stage (ASI; MS-50, LX-4000) to facilitate automated focusing. Both stages are controlled using the open-source Micro-Manager platform [23]. We note the inclusion of a Tungsten Halogen white light source and a beamsplitter in the illumination system, which enables us to easily switch between the modalities of traditional brightfield imaging, SA-DHM, and ODT.

The capture module is attached after the image plane of the microscope. This module is similar to the ‘X-module’ described in Ref. [22], with some small additions: the annular filter in the ‘X-module’ is replaced with a multi-pinhole filter and a photo-diode is added to facilitate alignment of the galvo-scanner with respect to these pinholes. In summary, the capture module has a 4f imaging system mapping the image plane to the detector plane making use of two identical lenses (Thorlabs UK; LB4282, 200 mm focal length). A polarising beamsplitter immediately after the first lens splits the wavefield into an object and reference that cross each other via mirror pairs (M3,M4) and (M5,M6) before being recombined using a 50/50 broadband cube beamsplitter (Thorlabs UK; BS031). The mirror pairs are aligned to produce a matching overlapping image on the detector from both paths (in the absence of F), each with an angle of incidence on the detector that is equal and opposite; these angles combined provide the off-axis condition as described in more detail in Ref. [22]. Two rotatable linear polarisers, P1 and P2 (Edmund Optics UK; LPVISE200-A), at the input and output of the module enable independent control of the object and reference power on the monochrome digital camera (Thorlabs; CS895MU), which has 4096 × 2160 pixels of size 3.45 × 3.45 μm . The 180 focused laser spots in the filter plane (FP) are reproduced at the back focal plane of the first lens in the reference path where these are aligned with 180 pinholes on a customised filter, (JD-Photo, UK). This filter, F, is mounted on three-dimensional translation stage (Thorlabs UK; DT12XYZ/M) and is composed of a 25 × 25 × 3 mm glass plate with a chrome photo-mask of optical density >3 produced using photo-lithography. The pinholes are located on a circle of 2.5 mm radius and have diameter 15 μm . The filter also includes a pinhole in the centre of the annulus to facilitate initial alignment. An identical piece of glass without photomask is inserted into the object path in order to maintain phase-matching between both paths. Following an alignment protocol, described below, the precise set of galvonometer voltages that align the laser focus with the 180 pinholes in the filter are known. The 180 holograms are recorded in sequence using a square-wave software trigger that steers the galvanometer to each pinhole, and synchronously controls the camera acquisition time to match the dwell time of the laser focus at each pinhole. The total time taken to record all holograms is 10s using an acquisition time of 40ms for each hologram.

## 3. Image formation

The laser focus is moved in the filter plane to 180 discrete position in a circle around the origin, where each unique position denoted by index n is given by $\left(p_{n},q_{n}\right)$ . The resulting oblique plane wave illumination in the sample plane is given by $\exp[jk(xp_{n}+yq_{n})]$ , where the wave vector k=2π/λ , $p_{n}=NA_{ill}\cos\left[2\pi /(n-1)\right]$ and $q_{n}=NA_{ill}\sin\left[2\pi /(n-1)\right]$ , and $NA_{ill}$ is the numerical aperture of the illumination that must satisfy $NA_{ill}\leq NA_{MO}$ . The resulting complex image, $t_{n}^{\prime }(x,y)$ , in the image plane of the microscope has a Fourier transform, $T_{n}^{\prime }(u_{x},u_{y})$ , defined in Eq. (1):

(1) $$T_{n}^{\prime }(u_{x},u_{y})=T(u_{x}-p_{n},u_{y}-q_{n})H(u_{x},u_{y})$$

where (x,y) and $\left(u_{x},u_{y}\right)$ denote the spatial and spatial frequency coordinates. $T(u_{x},u_{y})$ is the Fourier transform of the sample transmittance function, t(x,y) , and $H(u_{x},u_{y})$ is the transfer function of the microscope, which is defined as a pupil function of radius equal to $NA_{MO}/\lambda$ , where λ is the laser wavelength. For each illumination n , the resulting object and reference wavefields in the detector plane are given by:

(2) $$\begin{array}{l} O_{n}(x,y)=t_{n}^{\prime }(x,y)\exp[jk(\alpha x+\beta y)]\\ R_{n}(x,y)=\exp[jk(xp_{n}+yq_{n})+j\phi_{n}]\exp[-jk(\alpha x+\beta y)] \end{array}$$

The reference is produced by filtering the unscattered field using a pinhole at position $\left(p_{n},q_{n}\right)$ on the filter. The off-axis angles of the object and reference are opposite and equal and are related to α and β in the above expression. The phase term $\phi_{n}$ in the reference account for any small misalignment in the object and reference arms with respect to slight tilt of the filter or slight path length mismatch. The resulting hologram is given by:

(3) $$\begin{array}{l} hol_{n}(x,y)={\left|t_{n}^{\prime }(x,y)\right|}^{2}+1\\ +t_{n}^{\prime }(x,y)\exp[j2k(\alpha x+\beta y)]\exp[-jk(xp_{n}+yq_{n})-j\phi_{n}]\\ +\bar{t_{n}^{\prime }(x,y)}\exp[-j2k(\alpha x+\beta y)]\exp[jk(xp_{n}+yq_{n})+j\phi_{n}] \end{array}$$

which has a Fourier transform, $HOL_{n}$ , given by:

(4) $$\begin{array}{ll} HOL_{n}(u_{x},u_{y}) & =F\{|t_{n}^{\prime }(x,y){|}^{2}\}(u_{x},u_{y})+\delta (u_{x},u_{y})\\ & +\exp(-j\phi_{n})T_{n}^{\prime }(u_{x}+2\alpha +p_{n},u_{y}+2\beta +q_{n})\\ & +\exp(j\phi_{n})\bar{T_{n}^{\prime }(u_{x}-2\alpha -p_{n},u_{y}-2\beta -q_{n})} \end{array}$$

where F is the Fourier transform operator. This equation can be rewritten in terms of the original transmittance as follows:

(5) $$\begin{array}{ll} HOL_{n}(u_{x},u_{y}) & =F\{|t_{n}^{\prime }(x,y){|}^{2}\}(u_{x},u_{y})+\delta (u_{x},u_{y})\\ & +\exp(-j\phi_{n})T(u_{x}+2\alpha ,u_{y}+2\beta )\\ & \times \bar{H_{n}(u_{x}-2\alpha -p_{n},u_{y}-2\beta -q_{n})}\\ & +\exp(j\phi_{n})\bar{T(u_{x}-2\alpha ,u_{y}-2\beta )}\\ & \times H(u_{x}+2\alpha +p_{n},u_{y}+2\beta +q_{n}) \end{array}$$

Interestingly T does not move in the spatial frequency domain as the illumination varies, i.e. the zero-frequency position of T in the expression above remains static regardless of the illumination index n , which is the basis of real time SA-DHM [22]. This is a unique feature of our ODT implementation when compared with other systems.

## 4. Alignment protocol

The initial alignment is based on steering the galvo-scanner to focus at the centre of the filter plane, FP. In the absence of any filter or glass plate in the capture module, the mirrors are adjusted to produce an aligned image of an arbitrary sample in the detector plane for both paths. Maintaining image alignment for both paths, the mirrors are further adjusted to produce an in-line interference pattern, i.e. both paths are co-linear. Using both pairs of mirrors (M3,M3) and (M4,M6) the object and reference paths are ‘walked’ (in the context of ‘walking the beam’) to produce equal and opposite off-axis angles, always maintaining image alignment, such that a suitable off-axis condition is satisfied. The filter, F, and glass plate, G, are added to the capture module, and the former undergoes manual 3-D translation/tilt to optimise light transfer through the centre pinhole.

The second step in alignment is to determine the 180 discrete voltage pairs to be applied to the two galvonometers in order to achieve optimal light transfer through the 180 annular pinholes. This is achieved by raster-scanning in the approximate region of each pinhole (the approximate region being determined by geometry) and recording the intensity of the throughput using a photo-diode; the maximum value corresponds to the optimum steer angle. The entire process is automated through a LabVIEW Virtual Instrument (VI) that synchronously controls the galvanometers and reads outputs from the photodiode, utilizing a National Instruments USB-6000 DAQ. This automation, which completes in approximately 5 minutes, determines and returns the galvanometer voltages corresponding to each pinhole’s position. The frequency of this calibration step is dependent on environmental vibrations; for our system, conducting it daily proved sufficient.

Regarding sensitivity to vibration, although we made no attempt to measure the effect of vibration, we have found that the system is relatively robust to environmental vibration and was relatively noise free when compared with the traditional off-axis architecture for which the reference and object paths are entirely separate. It is likely that this results from the partially common path architecture, whereby the object and reference paths are identical prior to the X-module. It is likely that robustness to vibration could further be improved by adopting a fully common path architecture, which may be a possible avenue for future research.

## 5. Methods of numerical tomographic reconstruction

**Fig. 2.**
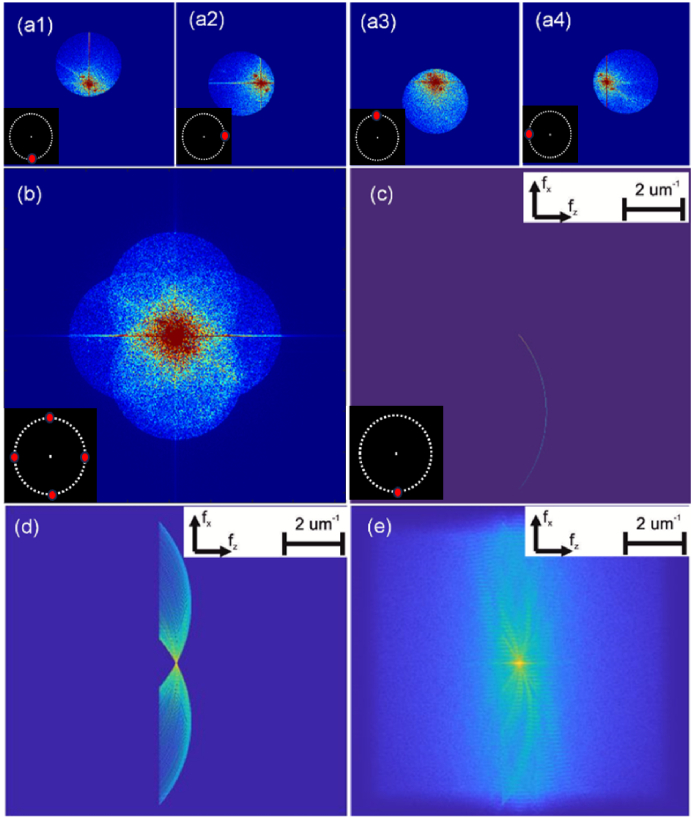
(a1-a4). The amplitude of four example spatial frequency apertures of the samples complex transmission function, T , captured using the module. These are produced by applying the DFT to four recorded holograms matching the illuminations indicated by the points on the annular filter shown on the left of each image. For each case, one of the complex twin image terms has been cropped, and the DC term and other twin are not shown. Note the aperture pupil function, $H_{n}$ , moves in an annular pattern in the DFT due to the self-reference design such that the zero-frequency for each capture is always co-located; (b) These four complex DFTs can be superimposed to achieve SA-DHM. This is implemented by either capturing the holograms independently and adding, or by strobing the illuminations quickly within the acquisition time of the camera. In the general case, all 180 holograms can be superimposed. (c) For ODT, each 2D-DFT is projected onto the 3D Ewald sphere in 3D Fourier space; a single case is shown in the figure. (d) All 180 twin image DFTs are projected on the Ewald sphere to produce F ; (e) The missing values are filled in by numerical processing, see text.

The *first step* in reconstruction is to compute the complex images for each illumination angle by filtering the twin image term in the DFT of each hologram as described in Ref. [22]. Four such filter twin image terms are shown in Fig. 2(a1-a4) for a sample described later in the text. As we will show later in the text, these four DFT patterns can be superimposed to achieve SA-DHM, either by recording the holograms independently and adding, or by strobing the different illuminations within the camera acquisition time. This is illustrated in Fig. 2(b). The *second step* for ODT is to reconstruct the 3D spectrum of the scattering potential function, $F(u_{x},u_{y},u_{z})$ by superimposing the 180 DFTs of the complex images each of which is mapped onto an Ewald sphere in 3D Fourier space. More specifically, we use the first order Rytov approximation to produce 180 Rytov phase terms: $\Psi_{n}(x,y)=\ln[t_{n}^{\prime }(x,y)/t_{n_{i}}(x,y)]$ where $t_{n_{i}}$ is the background complex image recorded using no sample. These are then mapped to the Ewald sphere as follows: $F_{n}(u_{x},u_{y},u_{z})=-\frac{j(u_{z}+u_{0}n_{0})}{\pi }F\{\Psi_{n}(x,y)\}$ , which are super-positioned to produce F ; the result of this process for a single projection is shown in Fig. 2(b) and for all 180 projections is shown in Fig. 2(c). Typically in ODT, the division of $t_{n_{i}}$ will reposition the twin images in 2D Fourier space such that the position of the zero spatial frequency of each is co-aligned for all cases. In our implementation this repositioning is achieved optically as a natural consequence of the self-reference design in which the the reference angle changes synchronously with the illumination angle as shown for four cases in Fig 2 (a1-a4) and (b). Phase matching of each of the DFTs is also required, $F\{\Psi_{n}\}$ . [24] such that the zero frequency components have the same phase value; this phase value can vary across the images due to small path length differences, i.e $\phi_{n}$ is not constant. Due to the limited illumination angle and numerical aperture there are large areas in F with missing information.

The *third step* is to employ an iterative non-negativity constraint algorithm, known as the total variation iterative constraint Gerchberg-Papoulis (TVIP-GP) algorithm to fill in the missing information. The TVIC algorithm [12] is a minimisation algorithm that is first applied to F , to generate a binary mask, M , that defines the spatial support of the specimen, which is then passed to the GP algorithm. The GP algorithm is based on four iteratively repeated steps where [13,25]:

(1)
$\tilde{F_{i}^{\prime }}=(1-\beta_{i}\Omega ){\tilde{F}}_{i-1}+\beta_{i}\Omega \tilde{F_{0}}$
(2)
$f_{i}=MF^{-1}(\tilde{F_{i}^{\prime }})$
(3)
$f_{i}^{\prime }=g^{-1}(P_{\beta_{i}}P_{+}R\{g(f_{i})\})$
(4)
$\tilde{F_{i}}=F(F_{i}^{\prime })$

where $\tilde{F_{0}}=F$ and $\tilde{F_{i}}$ represents the recovered spectrum of the scattering potential function from the i th iteration. Ω is a 3D binary mask to distinguish the known region relating to the mapping of Rytov phase values; $\beta_{i}$ is a relaxation parameter (with initial value of 1) that is gradually reduced during iterations according to ( $\beta_{i}=0.99\beta_{i-1}$ ); g is a function to recover the complex refractive index of the sample by subtracting the refractive index of the surrounding immersion medium and has inverse $g^{-1}$ . R is an operator to extract the real part of the complex function; $P_{+}$ is an operator to apply the nonnegative constraint and $P_{\beta_{i}}$ is an operator to multiply a coefficient of $1-\beta_{i}$ to the padded regions in order to suppress oversampling. We applied 200 iterations of the GP algorithm to generate the tomographic reconstruction presented in this paper and the final result, $\tilde{F_{200}}$ is shown in Fig. 2(e) for the sample described below.

## 6. Results

**Fig. 3.**
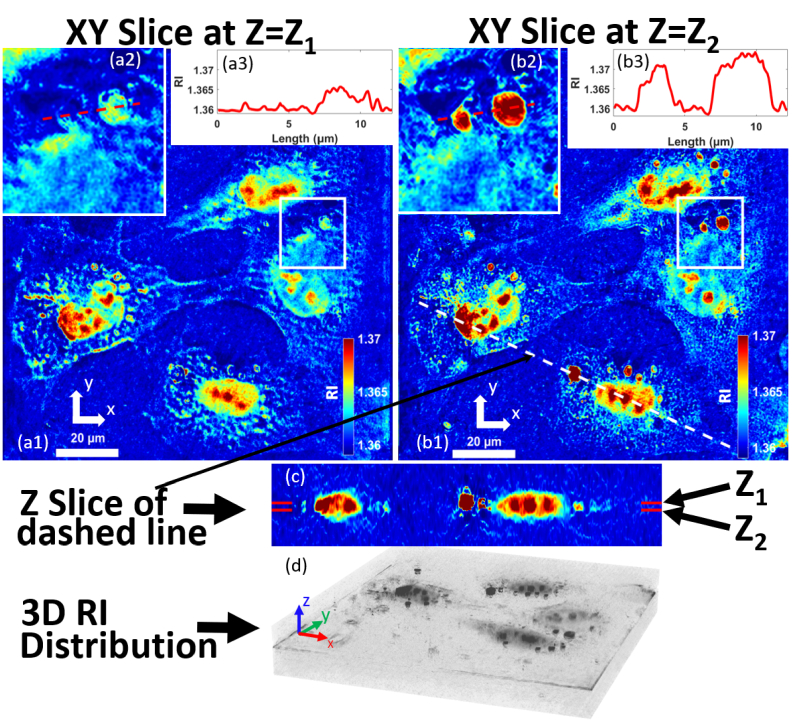
Tomographic reconstruction of cells; (a1-a3) and (b1-b3) show two different XY slices taken from the 3D Refractive Index distribution at two depths separated by 2.95 μ m. (a1) shows the RI distribution of the first slice, in which the square white box highlights a region that is shown magnified in (a2). A 2D plot of the RI distribution is shown in (a3) corresponding to the dashed red line highlighted in (a2). Corresponding figures are shown in (b1-b3) for an XY slice that is 2.95 μ m deeper in the RI distribution. (c) The vertical RI distribution corresponding to the dashed white highlighted in (b1). (d) Visualisation of the 3D refractive index distribution from which all of the earlier slices have been extracted,

Sample preparation is as follows: HEK cells were grown on a glass coverlslip and mounted on glass slide using FluorSave Reagent ( $n_{m}=1.36$ ). In total, 180 holograms were recorded using the module in approximately 10 seconds, and the reconstructed spectrum of the scattering potential function is shown in Fig. 2(c). From this, two slices of the x - y refractive index are extracted and shown in Fig. 3(a1) and (b1) with one slice centered in the cell nuclei and the other 2.95 μ m below. The same region in both images, highlighted by the white box, is magnified in Fig. 3(a2) and (b2) for comparison, in which it can be seen that there exist a number of small features, possibly lipid droplet formations, that are spatially resolved in the z direction. This is further emphasised using the profile of the refractive index across these feature shown in Fig. 3(b3) and (b3). A vertical z slice through the white dashed line shown in Fig. 3(b1), which cuts through two cell nuclei, is shown in Fig. 3(c); sub-cellular features are clearly resolved in the z direction. A point-cloud tomographic reconstruction is provided in Fig. 3(d).

## 7. Real-time synthetic aperture digital holographic microscopy

An important feature of the proposed module is that it can be used to implement a second modality, that of real-time SA-DHM [22], by extending the acquisition time of the camera to equal the time for each point source to move over all pinhole positions n:1→M , where in our case M=180 and recording a superposition hologram, which is the average of all of the individual holograms. This is similar to the system described in Ref. [22] except in that case continuous scanning of the laser focus was filtered using a continuous narrow annular filter in lieu of the pinhole filters used in this paper. Here, the acquisition time for the SA-DHM modality will be practically limited by the speed of the Galvo-scanner to steer the laser focus to each pinhole position and remain there for some short time. In this case, the single-capture SA-DHM hologram is given by:

(6) $$\begin{array}{ll} Hol & \left(u_{x},u_{y})=\sum_{n=1}^{M}Hol_{n}(u_{x},u_{y}\right)\\ & =M\delta (u_{x},u_{y})+\sum_{n=1}^{M}F\{|t_{n}^{\prime }(x,y){|}^{2}\}(u_{x},u_{y})\\ & +T(u_{x}+2\alpha ,u_{y}+2\beta )SA(u_{x},u_{y})\\ & +\bar{T(u_{x}-2\alpha ,u_{y}-2\beta )SA(u_{x},u_{y})} \end{array}$$

where,

(7) $$SA(u_{x},u_{y})=\sum_{n=1}^{M}\exp(-j\phi_{n})H(u_{x}+2\alpha +p_{n},u_{y}+2\beta +q_{n})$$

**Fig. 4.**
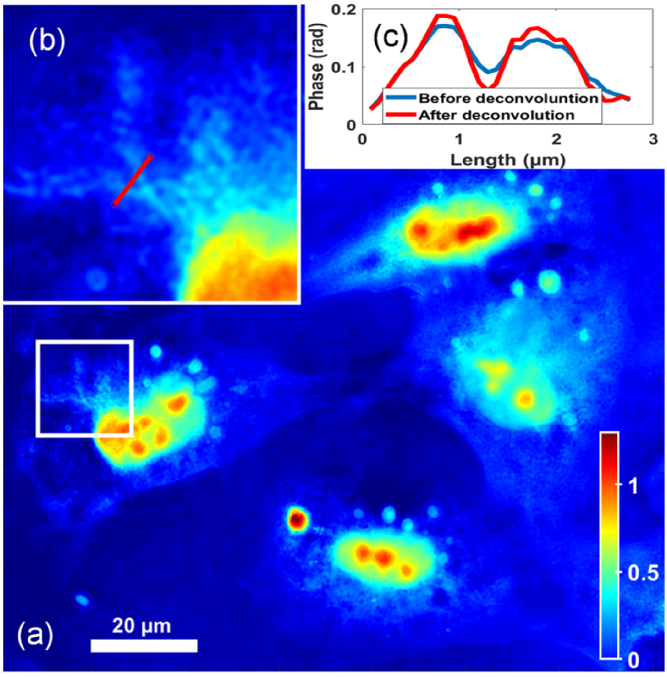
(a) Phase image using SA-DHM by adding the raw holograms; (b) a small region in magnified and a profile of the phase values over the red line is shown in (c).

The support of SA is given by $(NA_{MO}+NA_{ill})/\lambda$ . It is important that the laser is switched off during the transition of the laser focus from one fixed pinhole position to the next, in order to preserve the diffraction efficiency of the single capture SA-DHM hologram; if the laser remains active as the laser focus moves in between pinholes, no reference wave is incident on the camera while the object wavefield remains, and therefore, the DC term would be increased relative to the twin images. In order to avoid this, synchronisation of the galvo-scanner with a strobe laser using TTL-logic, or with a chopper, is required. This was beyond the capability of the laser diode used in our set-up, and for this reason, we simulate the real-time SA-DHM modality by adding together the 180 raw holograms that were captured for ODT and reconstructing the image using only two FFT algorithms. The result is given in Fig. 4(a). Despite the use of a coherent source to create the SA-DHM, there is no evidence of coherent noise in the image due to the averaging process. Since, the synthetic aperture profile can easily be determined from Eq. (7), it is possible to implement real-time non-blind deconvolution by multiplying the filtered twin image by the inverse of H (with a regularisation parameter) in between the two DFT operations; see Ref. [22] for more details. To highlight the improvement in the image contrast provided by deconvolution, the region in the white box is magnified and shown in Fig. 4(a) and a profile of a small sub-cellular feature is shown in Fig. 4(c) before and after deconvolution.

## 8. Resolution

The resolution of the final image will depend on the modality that is being used. For the case of ODT, the spatial and axial resolution of the 3D refractive index map are given by [26,27]:

(8) $$R_{lateral}=\frac{\lambda }{2(NA_{MO}+NA_{ill})}$$

(9) $$R_{axial}=\frac{\lambda }{2}\frac{1}{n_{i}-\sqrt{n_{i}^{2}-NA_{MO}^{2}}}$$

where $n_{i}$ is the refractive index of the immersion medium. For our setup, ( $n_{i}=1$ , $NA_{MO}=0.85,NA_{ill}=0.68NA_{MO}$ , the lateral and axial resolutions of the RI images are given by: $R_{lateral}=222$ nm and $R_{axial}=671$ nm. Using an oil-immersion ( $n_{i}=1.51$ ) objective of $NA_{MO}=1.3$ and the same relative value for $NA_{ill}$ would result in smaller values of $R_{lateral}=145$ nm and $R_{axial}=428$ nm. For the case of real-time SA-DHM, the spatial and axial resolution of the quantitative phase image is given by:

(10) $$R_{lateral}=\frac{\lambda }{NA_{MO}+NA_{ill}}$$

(11) $$R_{axial}=\frac{2\lambda }{NA_{MO}^{2}}$$

with the latter being related to the depth of field [28]. For our setup, the lateral and axial resolutions of the SA phase image are given by: $R_{lateral}=444$ nm and $R_{axial}=1.758$ μ m but would reduce to $R_{lateral}=290$ nm and $R_{axial}=752$ nm using an immersion objective of $NA_{MO}=1.3$ .

## 9. Conclusion

In this paper, we have proposed a new method for recording the 3D ODT tomographic image with several advantages over the state of the art. Firstly the system is modular and employs a self-reference design, such that it can easily be added to any existing life-science microscope and with relatively low cost; the alignment procedure is relatively straightforward and has been automated using a raster-scanning approach that takes approximately 5 minutes. Once aligned, the system records 180 holograms in 10 seconds but this could be reduced using a higher speed DAQ. Secondly, the method is capable of implementing real-time SA-DHM if a strobing laser diode is used. Although we do not experimentally demonstrate the real-time implementation, we simulate by simple superposition of the 180 raw holograms and a single DFT to reconstruct the image, which has low levels of coherent noise. We believe this modular bi-modal implementation of ODT/SA-DHM will be of practical use in life-science imaging.

## Data Availability

Data underlying the results presented in this paper are not publicly available at this time but may be obtained from the authors upon reasonable request.
